# Strain-Engineered Jacutingaite Analogs as Efficient 2D Catalysts for Hydrogen Evolution Reactions

**DOI:** 10.1021/acsomega.5c09065

**Published:** 2025-11-28

**Authors:** Caique C. Oliveira, Pedro A. S. Autreto

**Affiliations:** Center for Natural and Human Sciences (CCNH), 74362 Federal University of ABC (UFABC), 09210-170 Santo André, SP, Brazil

## Abstract

The catalytic properties of Pt_2_XSe_3_ (X = Hg, Zn) for Hydrogen Evolution Reactions (HER) have been investigated based on state-of-the-art ab initio simulations. Our findings indicate that the late transition metal sites (Hg and Zn) demonstrate superior activity for HER under acidic conditions. Moreover, lattice stretching or compression can significantly influence the H binding energy, achieving near-thermoneutral adsorption at a 3% compressive strain. This effect is attributed to the alterations in the d-band centers of late transition metal (X) sites and changes in the bonding strength, demonstrated by the changes in the integrated Crystal Orbital Hamilton Population (ICOHP). Furthermore, charge difference analysis reveals how charge accumulation between the X and Pt atoms changes as the structure is stretched (tensile strain), weakening the interactions with the H adsorbate due to the increased electrostatic repulsion. Our contribution explores strain engineering as an effective approach to tailor the catalytic activity of 2D materials for HER by providing insights into the role of mechanical manipulation in altering electronic properties and boosting catalytic performance.

## Graphical abstract

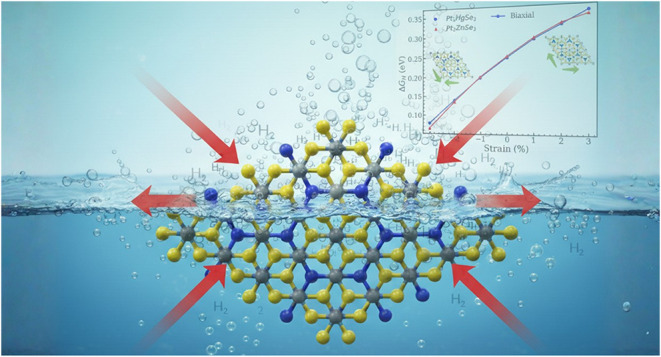

## Introduction

The increasing consumption of energy accentuates the need for clean, renewable, and efficient energy sources as viable alternatives to the diminishing reserves of fossil fuels that predominantly govern the global energy matrix.^1,2^ In this context, the advancement of strategic technologies, including batteries, supercapacitors,^3,4^ fuel cells, and electrolyzers, is critically important to facilitate the decarbonization of essential energy sectors.^5–7^ Hydrogen emerges as one of the most promising solutions owing to its high energy-to-mass ratio and its versatility for renewable energy production and storage.^8^ Green hydrogen, produced by water electrolysis employing clean and renewable energy sources (such as wind and solar) in the Hydrogen Evolution Reactions (HER),^9^ has attracted significant interest due to its inherently sustainable nature. High-performance electrolyzers typically utilize noble metal-based catalysts, notably platinum, which restricts their commercial viability because of the limited availability of these materials.^10^ Thus, minimizing the noble metal content is crucial in the development of economically viable catalysts.

Two-dimensional materials have been widely studied for catalysis applications.^11–13^ The high surface area and enhanced charge mobility facilitate electron transfer, thereby augmenting their catalytic properties. Transition Metal Dichalcogenides (TMDs) have similarly been investigated extensively within this context.^14^ For hydrogen evolution reactions, it has been previously demonstrated that the activity is more pronounced at edge sites as opposed to the basal plane.^15,16^ Conversely, the basal planes of polymorphic 1T TMDs exhibit greater catalytic activity compared to the more typical 2H phases.^17,18^ Furthermore, doping, defect creation, as well as phase and strain engineering, are established strategies that can effectively modulate the electronic structure of these materials, promoting their catalytic properties.^16,19^

Notably, strain engineering is widely employed to tailor the catalytic activity of 2D TMDs. Lattice expansion or contraction can be achieved through different strategies,^20^ including depositing the target material on flexible substrates that can be bent,^21^ or wrinkled,^22^ inducing strain on the deposited material. This technique has been successful applied to MoS_2_ achieving almost 3% strain.^23,24^ Moreover, synthesizing materials with slightly different lattice parameters (lattice mismatch) can effectively induce biaxial strain.^25^ For example, lateral heterostructures of MoS_2_ and WS_2_ wth 1.59% biaxial strain were synthesized by epitaxial growth.^26^ Wang and colleagues showed that CVD-grown MoS_2_ crystals on SiO_2_/Si substrates can achieve a biaxial strain of 0.45%.^27^ Larger biaxial strains can be obtained by the pressure difference method. For example, Lloyd and colleagues reported 5.6% biaxial strain on MoS_2_ by depositing the material on a spherical cavity.^28^ Yang and colleagues have explored the effects of strain on the electronic, catalytic, and optical properties of MoS_2_/ZnO heterojunctions, which showed significant hydrogen evolution improvement under 5% compressive strain with Δ*G*_H_ of −0.04 eV.^25^ Lee and his team demonstrated that strain could induce thermoneutral hydrogen binding in PtSe_2_ flakes reaching a Δ*G*_H_ of 0.01 eV at the Se edge sites under 8% tensile strain.^22^ In addition, Lee and collaborators reported on the phase transformation in MoTe_2_ from the 2H to the 1T phase, inducing phase boundaries with internal strain that promoted their catalytic activity.^29^

Jacutingaite (chemical formula Pt_2_HgSe_3_, Figure 1a), is a naturally occurring compound originally isolated from the Caue mine in Itabira, Minas Gerais, Brazil.^30,31^ In addition to its natural occurrence, this compound has also been previously synthesized,^32–34^ garnering significant interest due to its exotic electronic properties. Lima et al. have methodically examined the stability of the Jacutingaite family, a group of materials characterized by the general formula M_2_XN_3_, where M represents Pt, Pd, and Ni, X denotes Zn, Hg, and Cd, and N corresponds to S, Se, and Te, all of which exhibit high stability. Furthermore, Pt and Pd-based structures were found to exhibit nontrivial topological behavior, as evidenced by the Topological invariant (*Z*_2_).^35^ Despite the growing interest in these materials, most of the current literature focuses on electronic,^36^ thermoelectric,^37^ topological^35,38^ and quantum^34,39,40^ properties, and a comprehensive assessment of its catalytic properties remains elusive. In this study, we examine the effects of X site substitution from Hg to Zn on the catalytic properties of the Pt_2_XSe_3_ structures in hydrogen evolution reactions in acidic conditions. We also explore how biaxial strain impacts the catalytic properties of these nanostructures by evaluating the changes in the H binding strength (Volmer step) as a function of in-plane deformations.

**Figure 1:**
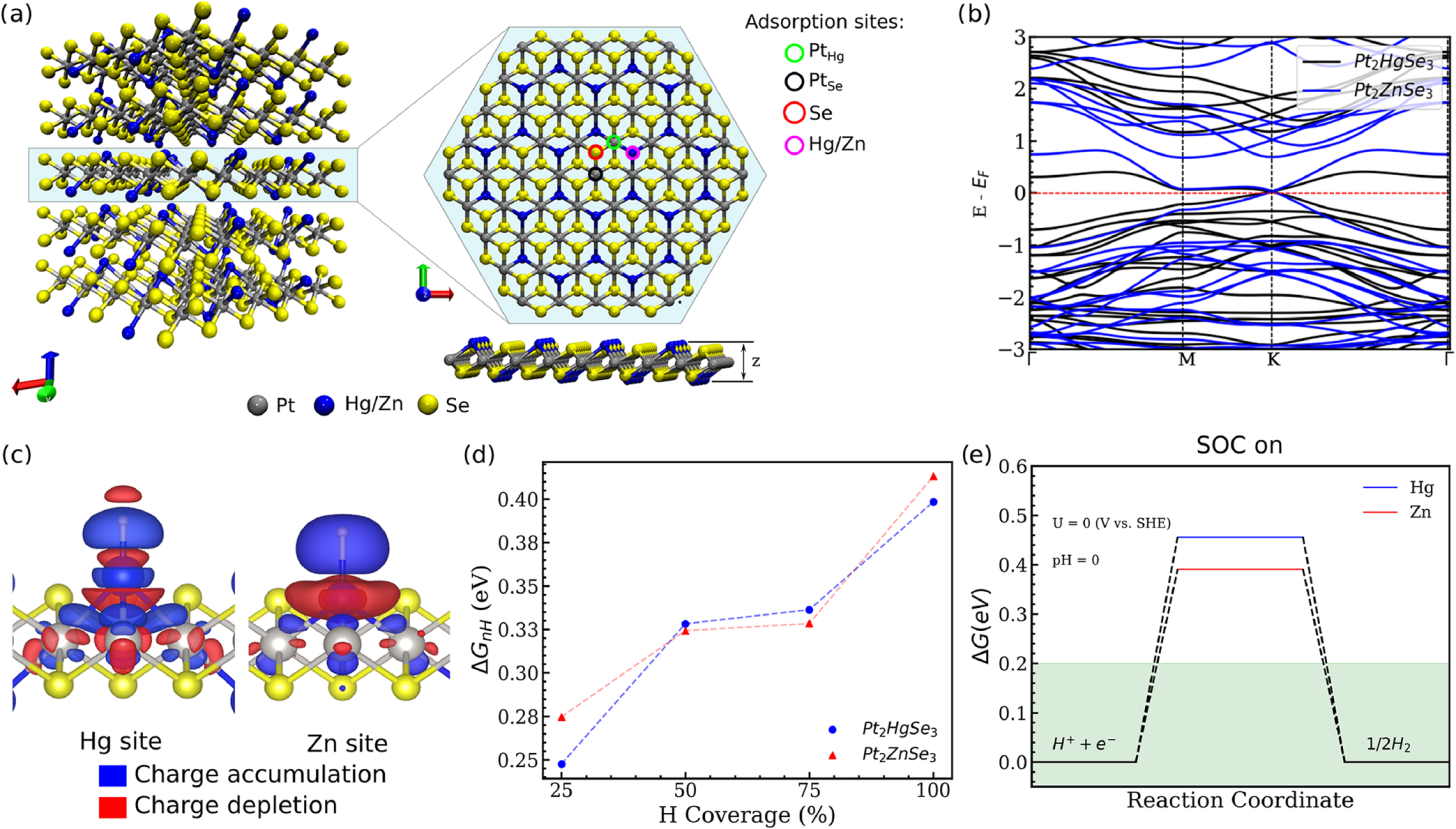
(a) Pt_2_XSe_3_ structures showcasing the monolayer and H adsorption sites. (b) Electronic band structure for the Pt_2_ZSe_3_. (c) Charge difference plots (Δ*ρ*) for the H adsorbed on Hg and Zn site at θ = 25%. Isosurfaces were set to 1.25 × 10^–3^ e/Å^3^. (d) Free energy as a function of the H concentration (θ). (e) H adsorption free energy diagrams for SOC calculations.

## Computational Details

Spin-polarized first-principles calculations were carried out using the *Vienna Ab-initio Simulation Package* (VASP).^41^ Electron–ion interactions were approximated using the Projected Augmented Wave (PAW) method.^42^ The following valence configurations were considered: 5d^9^, 6s^1^ for Pt; 5d^9^, 6s^2^ for Hg; 3d^10^, 4s^2^ for Zn and 4s^1^, 4p^4^ for Se. The exchange and correlation interactions were addressed through the Perdew, Burke, and Erzerhoff parametrization of the generalized gradient approximation,^43^ incorporating London dispersion interactions via the Becke-Johnson damping function^44^ as implemented in the DFT-D3 functional.^45^ Kohn–Sham orbitals were expanded using a plane-wave basis set, with a kinetic energy cutoff of 400 eV. The sampling of the Brillouin Zone (BZ) was conducted on a uniform *k*-point grid following the Monkhorst and Pack scheme.^46^ Calculations were performed on a 2 × 2x1 supercell with lattice parameters of 14.81 and 14.75 Å for Pt_2_HgSe_3_ and Pt_2_ZnSe_3_, respectively. Due to the quite big in-plane dimensions, a lower *k*-point mesh of 2 × 2 × 1 was employed for structural minimization, while for electronic structure calculations, which require a refined sample of the first Brillouin zone, an 8 × 8 × 1 grid was used. Throughout the simulations, a self-consistency threshold of 10^–7^ was maintained, with forces minimized below 0.02 eV/Å. To minimize interaction between the monolayer images, a vacuum of 15 Å was considered in the *z* direction. Data postprocessing was performed using the VASPKIT suite.^47^ Bader charges were calculated using the Henkelman package.^48^ The Crystal Orbital Hamilton Population (COHP) analysis^49^ was carried out to investigate the bonding nature of the H intermediate sites using the LOBSTER package.^50^

The elastic constants of the Pt_2_XSe_3_ structures were calculated using the energy-strain method,^51^ performing calculations of uniaxial and biaxial strain within ± 3% range in steps of 1%, according to the implementation of VASPKIT.^47^ For small elastic deformations, the energy of the system can be written as

1 $$E=E_{0}+\frac{1}{2}A_{0}\sum_{i,j=0}^{6}C_{\mathrm{ij}}\varepsilon_{i}\varepsilon_{j}+O\left(\varepsilon^{3}\right)$$

where *A*_0_ represents the area of the relaxed structure. For a 2D hexagonal system, the stiffness tensor (**C**) is given by

2 $$C=\left(\begin{array}{lll} C_{11} & C_{12} & 0\\ C_{12} & C_{11} & 0\\ 0 & 0 & C_{66} \end{array}\right)$$

where *C*_66_ is given by $\frac{1}{2}\left(C_{11}-C_{12}\right)$ . The independent elastic constants (*C*_11_ and *C*_12_) are obtained from the derivatives of eq 1,^52^ where the second derivative of *E* in the case of uniaxial strain (ε*_x_*) is related to *C*_11_ by

3 $$\frac{1}{A_{0}}\left(\frac{\partial^{2}E}{\partial \varepsilon_{x}^{2}}\right)=C_{11}$$

and the biaxial case (ε_*x*_ = ε_*y*_) gives

4 $$\frac{1}{A_{0}}\left(\frac{\partial^{2}E}{\partial \varepsilon_{x}\partial \varepsilon_{y}}\right)=2\left(C_{11}+C_{12}\right)$$

In acidic environment, the HER corresponds to an electrochemical reaction combining three steps: the initial reduction of a proton (H^+^) forming the adsorbed intermediate H (H^+^ + e^–^ → H*) is known as the Volmer Step; the subsequent formation of the H_2_ molecule by an electrochemical route, where the H* interacts with another H^+^, forming the molecule of H_2_ by one electron transfer (H* + H^+^ + e^–^ → H_2_) represents the Heyrovsky step. The formation of the H_2_ molecule can also proceed by a chemical route, where two neighboring H* interact to form a H_2_ (2H* → H_2_) representing the Tafel step.^9^ The catalytic activity of the structures was assessed by computing the H adsorption free energy (Δ*G*_H*_) employing the Computational Hydrogen Electrode (CHE) model of Nørskov and co-workers,^53^ calculated as follows

5 $$\Delta G_{H*}=\Delta E_{nH*}^{\mathrm{ads}}+\Delta E_{\mathrm{ZPE}}-T\Delta S$$

The adsorption energy for *n* intermediates is calculated by the equation:^54^

6 $$\Delta E_{nH*}^{\mathrm{ads}}=E_{nH*}-\left(E_{\left(n-1\right)H*}+\frac{1}{2}E_{H_{2}}\right)$$

where *E*_*n*H*_ represents the total energy of the structure with an *n* adsorbed H, *E*_(*n*–1)H*_ the total energy of the structure with (*n*–1) adsorbed H adsorbates, and *E*_H_2__ the total energy of a H_2_ molecule (in gas phase, p_H_2__ = 1 bar) and T the absolute temperature (298.15 K employed in this work). Δ*E*_ZPE_ and Δ*S* are the zero-point energy and entropic variation with respect to H_2_ gaseous phase. Following previous works, these last two terms are approximated by 0.24 eV.^55^ In general, good catalysts exhibit thermoneutral H binding with Δ*G*_H_ ≈ 0, strong enough to form adsorbed H intermediates (H*) without harnessing the desorption of products (H_2_). The d-band center (ϵ_d_)^56^ was calculated using the following equation

7 $$\epsilon_{d}=\frac{\int_{-\infty }^{E_{F}}\mathrm{ED}\left(E\right)dE}{\int_{-\infty }^{E_{F}}D\left(E\right)dE}$$

where *D*(*E*) represents the d-band density of states at the *E* energy eigenvalue. Differential charge density analysis was carried out to investigate the charge rearrangement after the adsorption process according to the following

8 $$\Delta \rho =\rho_{H*}-\rho_{*}+\rho_{H}$$

where ρ_H*_, ρ_*_, and ρ_H_ represent the self-consistent charge density of the optimized slab with adsorbed H, the optimized slab, and the bare adsorbate, respectively.

## Results and Discussion

Jacutingaite is a van der Waals-layered compound, structurally similar to sudovikovite (PtSe_2_).^31^ The structure is modified in such a way that one-fourth of the chalcogen atoms are substituted by a late transition metal: Zn, Hg, or Cd, forming a sublattice of hexagons as shown in the top view of a monolayer depicted in Figure 1a. Therefore, the platinum (Pt) atoms can be either coordinated with four selenium and two late metal atoms (Hg or Zn) atoms or coordinated with six selenium (Se) atoms. The different coordination gives rise to a buckling height (*z*, depicted in Figure 1a), since the bond lengths are expected to change in Pt–Se and Pt–Hg/Zn-Se octahedra. The optimized lattice parameters are *a* = *b* = 7.50 Å with a buckling height (*z*) of 3.50 Å for Pt_2_HgSe_3_ and *a* = *b* = 7.46 Å with *z* = 2.72 Å for Pt_2_ZnSe_3_, in good agreement with previous studies.^35,57^ The electronic band structure is shown in Figure 1b, from which it can be seen that the modification of the X species does not change the overall electronic behavior of the structure. In other words, the nonzero gap character is conserved, and the Dirac-like feature at the *K* point is preserved. This result is in good agreement with previous calculations using DFT.^35^ Analyzing carefully, one can notice that the behavior of the valence and conduction bands is similar near the *K* high-symmetry point, and the dispersion relation becomes very different toward the Γ point: for Zn, it seems that the band broadens in this direction. Furthermore, for Hg, there is a gap in the conduction region right above the first conduction band, whereas for Zn, this gap is smaller. Generally speaking, if one disregards the last (first) valence (conduction) bands, when X = Zn, the bands shift to lower energy values.

The catalytic activity of both structures was investigated by calculating the H adsorption free-energy (Δ*G*_H*_) for the nonequivalent sites of the Pt_2_XSe_3_ monolayers. In total, four sites were considered in each structure: the top Se (Se) sites and the top late transition metal (Hg or Zn) sites. Also, the two distinct coordination environments for Pt have been taken into account: the Pt_Se_ sites represent the case where the noble metal is coordinated with 6 Se, whereas the Pt_Hg/Zn_ corresponds to the octahedra containing two X and 4 Se atoms. A representative of each site is illustrated in Figure 1a. The corresponding Δ*G*_H*_ values are presented in Table 1 where it can be seen that only the Se site on the Pt_2_HgSe_3_ and the X sites (Hg or Zn) have interesting results. In particular, the Hg and Zn sites show promising results with Δ*G*_H_ of +0.24 and +0.27 eV, very close to the optimal adsorption range (|Δ*G*_H*_| < 0.20 eV) respectively.^58^ For the other cases, Se sites on the Pt_2_ZnSe_3_ show weak binding, whereas in the Pt_X_ sites, the H migrates to the center of the hollow site formed by the chalcogen and late transition metals, interacting more with these atoms as shown by the charge difference analysis presented in Figure S1 of the Supporting Information (SI). The differential charge analysis for the Hg and Zn sites is presented in Figure 1c. This analysis reveals charge accumulation around the H intermediates. Charge depletion is located right above and below the Hg atom. Furthermore, significant charge accumulation is observed between the Pt and Hg atoms. For the Zn site, charge also accumulates on H, and the depletion on the Zn atom is uniform, in contrast with Hg, where charge depletion is located on the poles. As both isosurfaces are the same, more charge rearrangement is observed for Zn compared to Hg. Also, less charge accumulation is observed between Zn and Pt atoms, balancing the larger charge located on the H.

**Table 1:** H Adsorption Free Energies on Non-Equivalent Sites of the Pt_2_XSe_3_ Structures

structure	site	Δ*G*_H*_ (eV)
Pt_2_HgSe_3_	Pt_Se_	0.89
	Pt_Hg_	1.00
	Se	0.66
	Hg	0.25
Pt_2_ZnSe_3_	Pt_Se_	2.47
	Pt_Zn_	2.21
	Se	3.43
	Zn	0.27

We have also investigated the effect of H coverage on the H adsorption free energy as shown in Figure 1d. The different H coverages were simulated by occupying one-fourth (25%), half (50%), three-fourths (75%), and all the Hg and Zn sites on one side of the Pt_2_XSe_3_ monolayers, as shown in Figure S2 of SI. The results reveal that the adsorption strength is weakened by the increased amount of H adsorbed, in agreement with previous studies of H adsorbed on the basal plane of TMDs.^59^ This result can be attributed to the structural deformations induced by the increased amount of adsorbates and their repulsion, increasing the Δ*G*_*n*H*_. Given that Pt_2_XSe_3_ structures are recognized for significant spin–orbit coupling (SOC) effects,^35^ we examined SOC’s impact on the H adsorption free energy. Due to the computational complexity, these calculations were conducted on a unit cell, equating to 100% X site coverage. Figure 1e displays the results, with Δ*G*_H_ values of 0.46 eV for Hg and 0.39 eV for Zn, aligning closely with non-SOC values (0.40 and 0.41, respectively). Notably, the Hg site deviates significantly (almost 18%) from non-SOC results, prompting questions on whether heavier elements, susceptible to SOC effects, could markedly alter catalytic properties. However, a detailed exploration of these effects is outside the scope of this study.

The results presented thus far suggest that replacing Hg with Zn retains most of the material’s electronic and catalytic characteristics. However, structural properties–such as lattice parameters and buckling height–differ, which is expected due to the distinct atomic properties of each element, including atomic and van der Waals radii, as well as electronic configuration (Hg contains 4f electrons, while Zn has only 3d). Nevertheless, the similarity in key properties supports the substitution with Zn, which poses less environmental harm compared to Hg.

Biaxial strain has been shown to effectively modulate the HER intermediates adsorption free energies by regulating the electronic states.^60,61^ Moreover, large percentages of biaxial strain was achieved by depositing highly impermeable 2D MoS_2_ on suspended membranes, which, by pressure difference applied on the latter, induce biaxial deformation on the deposited 2D TMD.^28^ Zhang and colleagues have proposed a new “in situ self-vulcanization strategy” where biaxially strained MoS_2_ nanoshells that encapsulate Ni_3_S_2_ (a core–shell heterostructure) were obtained with 5% biaxial strain with improved HER activity.^62^ Drawing from these studies here we investigate the effects of biaxial strain on the catalytic properties of the Pt_2_XSe_3_ structures. The strain (ε) is defined as

9 $$\varepsilon \left(\%\right)=\frac{\left(a-a_{0}\right)}{a_{0}}\times 100$$

where *a*_0_ represents the equilibrium lattice constant and *a* the deformed lattice constant. For each value of strain, the atoms in the structure are allowed to relax with fixed lattice constraints. In this work, biaxial strain was achieved by simultaneously stretching/compressing *a* and *b* lattice vectors in within the range of ± 3% in steps of 1%, ensuring that the deformations on the structures are in the elastic regime, indicated by the strain energy (*E*_strain_) defined as^25^

10 $$E_{\mathrm{strain}}=E\left(\varepsilon \right)-E_{0}$$

where *E*(ε) represent the total energy of the strained system and *E*_0_ the total energy of the unstrained structures. As shown in Figure 2a, biaxial deformations within 3% result in a quadratic behavior for *E*_strain_, indicating that deformations within this range are reversible and, therefore, the elastic regime is conserved.^25^ We have also calculated the elastic constants of the Pt_2_HgSe_3_ and Pt_2_ZnSe_3_ structures using the procedures described in the Computational Detailssection. The obtained elastic constants, Young modulus (γ^2D^) and Poisson’s ratio (ν) are presented in Table 2, from both criteria (*C*_11_ > 0 and *C*_11_ > *C*_12_) are satisfied.^63^ The Pt_2_ZnSe_3_ structure presents slightly higher γ^2D^, indicating that it is stiffer compared to the Pt_2_HgSe_3_ counterpart. Interestingly, the Poisson ratio for Pt_2_HgSe_3_ 2D nanostructure is very close to the reported value of 0.33 (calculated with Voigt averages) for the bulk counterpart.^64^

**Figure 2:**
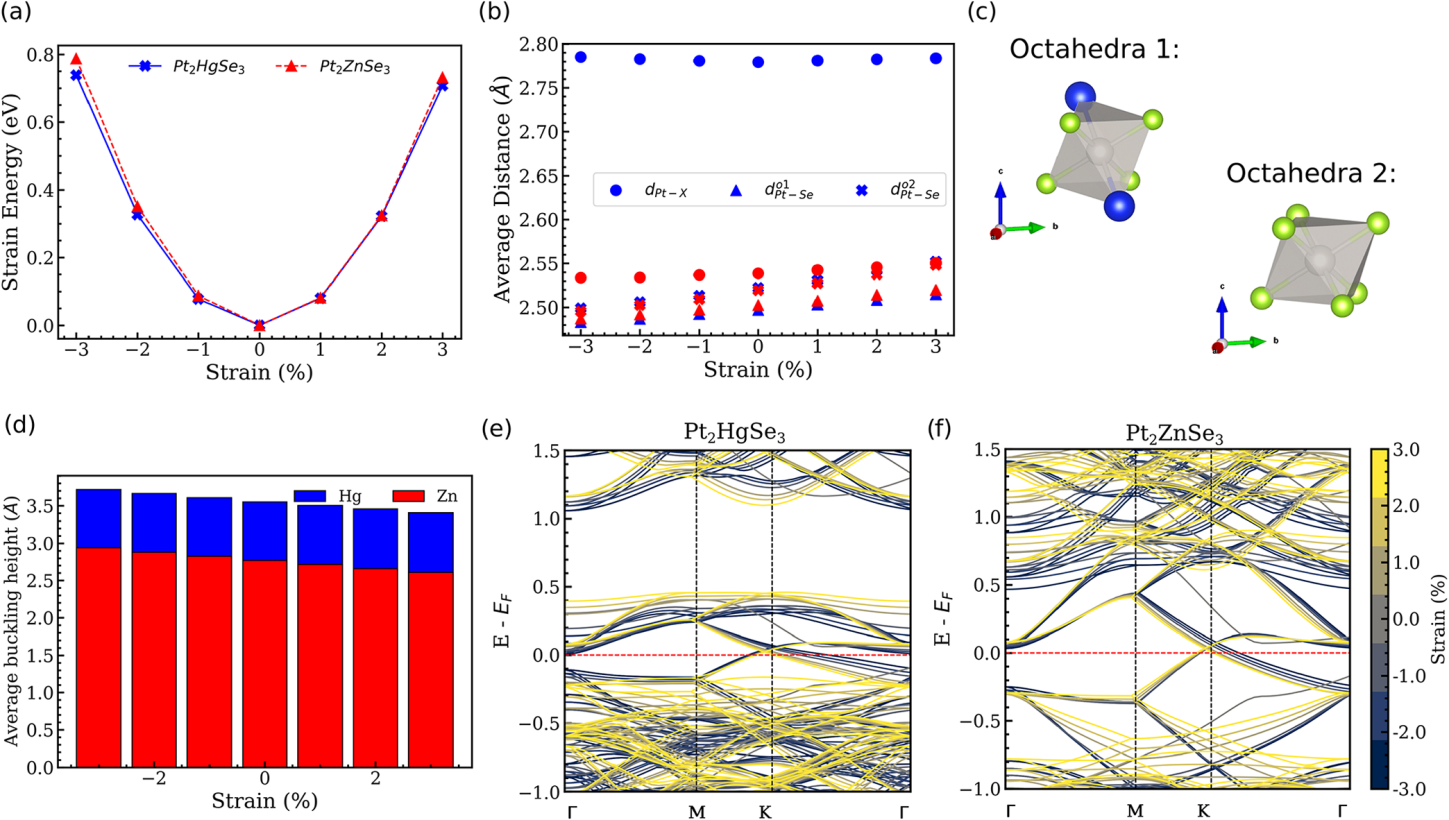
(a) Total energy relative to the equilibrium (strain energy). (b) Average distances between Pt and Hg/Zn atoms *d*_Pt–X_ and between Pt and Se atoms on the two octahedra present in the structures (c). (d) Average buckling height (*z*) as a function of the strain. The electronic band structures as a function of the strain for (e) Pt_2_HgSe_3_ and (f) Pt_2_ZnSe_3_.

**Table 2:** Obtained Elastic Constants, Young Modulus (γ^2D^) and Poisson’s Ration (ν) for the Pt_2_XSe_3_ 2D Structures

structure	*C*_11_ (N/m)	*C*_12_ (N/m)	γ^2D^ (N/m)	ν
Pt_2_HgSe_3_	52.52	16.76	41.18	0.32
Pt_2_ZnSe_3_	54.25	18.61	47.87	0.34

Regarding the structural changes, we analyze the distortions in the two octahedra (presented in Figure 2c): one formed by the Pt atom coordinated with four Se atoms and two late transition metals (X) (octahedra 1), and the other formed by the Pt atom coordinated with eight Se atoms. Figure 2b presents the average distances between Pt and Se (*d*_Pt–Se_^*o*1^), Pt and X (*d*_Pt–X_) in octahedra 1, and the distances between Pt and Se in octahedra 2 (*d*_Pt–Se_^*o*2^). Overall, the average distances between Pt and the late transition metal do not change significantly with strain, indicating that these bonds are less flexible. Contrarily, *d*_Pt–Se_^*o*1^ and *d*_Pt–Se_^*o*2^ follow an interesting pattern: at −3% compression, all the distances between Pt and Se are closer. As the structure is stretched, these distances increase. Our results show that *d*_Pt–Se_^*o*2^ is consistently larger than *d*_Pt–Se_^*o*1^, confirming that Se atoms are slightly closer to Pt when this atom is coordinated with two late transition metals. Moreover, *d*_Pt–Se_^*o*1^ follows almost a linear relation with respect to the applied strain. On the other hand, *d*_Pt–Se_^*o*2^ seems to increase at a higher rate, indicating that octahedra 2 is more flexible. We have also investigated the changes in the buckling height of the structure as a function of the applied strain. As shown in Figure 2d, when the Pt_2_XSe_3_ structures are compressed, the buckling increases, and when they are stretched, the buckling decreases. This behavior can be understood in terms of the previous discussions involving the two types of tetrahedra: compression elongates the octahedra in the direction of the late metal (Hg and Zn) atoms. On the other hand, when the structure is stretched, the octahedra are compressed. This observation aligns with the observed behavior for *d*_Pt–X_ shown previously, although this behavior is less evident for Hg. Mechanical deformations can also influence the electronic properties of 2D TMDs.^20,65^ Here, we have investigated the effects of biaxial strain on the electronic band structure of the Pt_2_XSe_3_. As shown in Figure 2e,f, large changes on the dispersion relations are observed for both Hg and Zn. In particular, at the Γ compression (expansion) raises (lowers) the bands. The same is observed for the band split at the *M* point, although the effects of strain can be well observed at the *K* point: for the higher band (originated from the splitting at *M*), the dispersion is raised with compression, while the lower bands are lowered. Interestingly, these effects seem to be similar for both Hg and Zn. As the electronic properties of the Pt_2_XSe_3_ structures are affected by strain, one could expect changes in their catalytic properties, as discussed next.

The results for the Δ*G*_H*_ as a function of strain are presented in Figure 3a (the values are presented in Tables S1 and S2 of the SI), where one can readily see that the adsorption strength of the intermediate can be effectively modulated when the structure is under elastic deformation. For instance, tensile strain weakens the interaction, resulting in an upshift in the Δ*G*_H*_. On the other hand, compressive strain results in stronger adsorption, modulating the Δ*G*_H*_ toward zero. Biaxial strain effectively modulates the H adsorption strength, given that at 3% compressive strain results in an adsorption free energy of 0.07 and 0.06 eV. The presented results underscore how biaxial strain can effectively modulate the binding strength of H intermediates, thus improving the catalytic activity of the structures. Our results can be summarized as follows: compressive biaxial strain is found to increase the catalytic activity of Pt_2_XSe_3_ nanostructures by increasing the H binding strength. Although these results are in contrast with those reported in early TMDs (such as MoS_2_ ^66^), where compressive strain is found to higher the Δ*G*_H_, our results are in agreement with the tendencies reported for late transition metals such as Cu.^67–69^ This discrepancy in the effect of compression strain stems from the electronic distribution of each transition metal, influencing how the electronic states on the d level, as discussed in the following.

**Figure 3:**
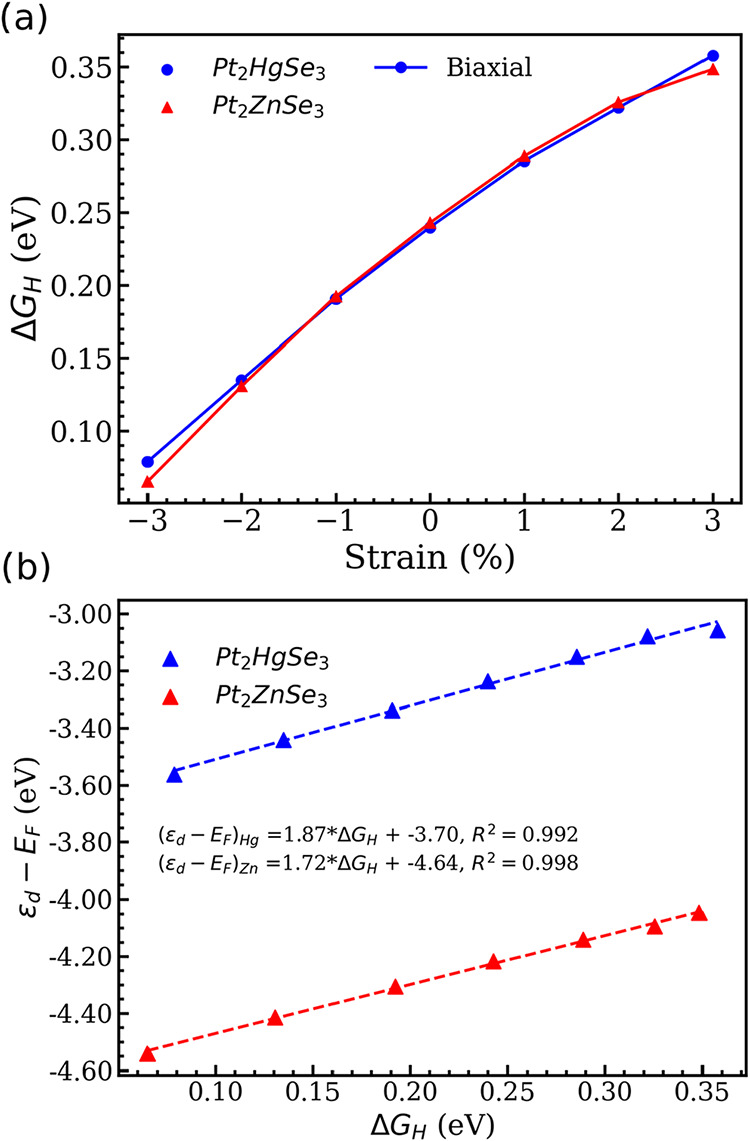
(a) Δ*G*_H_ as a function of strain for Pt_2_XSe_3_. (b) d-band center relative to the Fermi level (ε_d_) as a function of the Δ*G*_H_. The dashed lines represent the fit to the data, and the corresponding equations and *R* ^2^ are also presented.

To unravel the mechanisms underlying the activity changes as a function of elastic deformations, we have analyzed structural and electronic factors. Figure S3a of the SI shows the distance of adsorbed H and the active site as a function of applied strain. It can be seen that compression tends to approximate the adsorbate and active site, while expansion increases this distance. We have also calculated the Bader charges on the metal site (X = Hg or Zn) as a function of strain, as shown in Figure S3b of the SI. Overall, the charge on the metal sites changes within 0.05 |*e*| for biaxial strains on both Hg and Zn sites. Compressive strain of 1% decreases the charge to −0.60 (−0.30) on Zn (Hg), indicating improved charge transfer to the H intermediate. On the other hand 1% tensile strain shows the opposite trend, increasing the charge on the metal site, consistent with charge depletion from the intermediate. Further compression (tensile) does not reveal a clear trend, as the values fluctuate around the unstrained charges. These results indicate that mechanical deformation does not change the charges significantly, indicating that the catalytic activity changes also cannot be entirely described by this variable.

Previous studies have indicated that the modulation of Δ*G*_H*_ by strain is related to the optimization of the d-band center (ε_d_) in transition metal-based compounds.^22^ The relation between ε_d_ and Δ*G*_H_ is shown in Figure 3b where one can readily see that the d-band center with respect to the Fermi level (ε_d_ – *E*_F_) correlates linearly with the H adsorption free energy. This result is supported by the determination coefficient obtained from the fit (*R* ^2^), which is superior to 0.99 for both Pt_2_HgSe_3_ and Pt_2_ZnSe_3_. These two variables correlate as follows: for lower Δ*G*_H_ (compressive strain) lowers the ε_d_ moves away from *E*_F_, while the inverse behavior is observed for larger Δ*G*_H_ (tensile strain). An interesting result is that the calculated values for ε_d_ – *E*_F_ for Hg (left *y* axis in Figure 4b) and Zn (right *y* axis in Figure 4b) differ by ≈ 1 eV. The modulation of the ε with strain is well-known in literature:^67^ for late transition metals (more than half-filled d-bands), the width of the d-band becomes narrower (wider) as the structure is compressed (stretched) and to maintain the same level of filling, ϵ_d_ shifts downward (upward) resulting in the observed trend.

**Figure 4:**
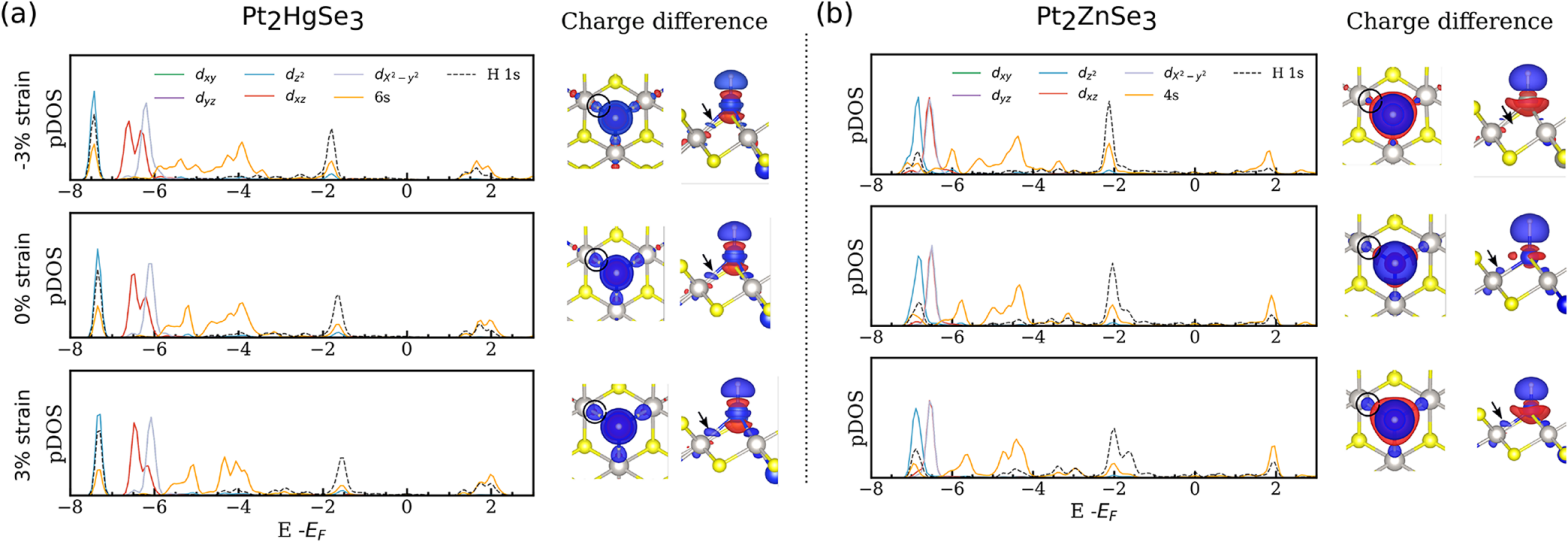
Projected density of states on the d orbitals, outer s and H 1s states and corresponding charge density difference analysis for −3, 0 and 3% strain for (a) Pt_2_HgSe_3_ and (b) Pt_2_ZnSe_3_.

Density of States (DOS) analysis is essential to explore the electronic changes resulting from mechanical deformations and from the interaction of the catalyst and the intermediates.^70,71^ Here, we analyzed the projected Density of States on the 5d (3d) and 6s (4s) orbitals of Hg (Zn) and H 1s states shown in Figure 4a,b. For Hg at 0% strain, there is a superposition between 5d_*z* ^2^_, 6s orbitals with H 1s at −8 and −1.75 eV below *E*_F_. On the other hand, for Zn these peaks are located at −7 and −2 eV below *E*_F_. The significant overlap between these orbitals indicates the hybridization between them. Lattice expansion causes depletion of the prominent peak at −2 eV below *E*_F_, and the same trend is observed for Zn, further corroborating the equivalence between the electronic and catalytic properties of these two elements. The depletion in the 6s (4s) orbitals indicates the decrease in the electron population in the bonding states, corroborated by the change in the integrated Crystal Orbital Hamilton Population (ICOHP) shown in Figure S4 of the SI further contributing to the destabilization of the bond between the H and the metal sites, thus explaining the weaker adsorption. Furthermore, the evolution of differential charge analysis for Hg (Figure 4a) and Zn (Figure 4b) also reveals an interesting result, where the charge accumulation between Hg (or Zn) and Pt atoms decreases (increases) when the structure is compressed (stretched). As less charge accumulation is observed in these regions, less electrostatic repulsion should be expected, and therefore, the bond strength should be higher. These results also corroborate the previous analysis explaining the intricate relation between relationship between strain and the catalytic activity, reflected by the changes in the Δ*G*_H_. Our findings contribute to the elucidation of how mechanical deformations (biaxial strain) can modulate the electronic structure of nanomaterials, improving their catalytic activity in HER which was also reported for 2D MoS_2_ in alkaline medium.^61,62^ Lattice strain was also found to optimize the electronic structure of Ce-based catalysts, where Y and Co codoping facilitated oxygen vacancy formation, inducing lattice contraction (compressive strain), significantly boosting activity for alkaline HER by reducing *H*_2_ *O* dissociation barriers.^72^ Activity improvement was also reported for Phthalocyanine and Polyoxometalate@carbon-nanotube heterostructures, where curvature-induced biaxial strain efficiently improved Oxygen Reduction Reaction catalysis.^73^

## Conclusions

In this work, we have conducted ab initio simulations based on DFT to investigate the catalytic properties of Pt_2_XSe3 (X = Hg, Zn) in HERs. The substitution of Hg by Zn results in the same overall electronic and catalytic behavior, given the similar electronic distribution of both elements. The late transition metal sites exhibit the best activity of HER at pH = 0, U = 0 at the SHE scale. Strain engineering effectively modulates the electronic properties of the Pt_2_XSe_3_ in a similar manner, indicating that the substitution of Hg by Zn, a more sustainable element, results in similar electronic behavior. Moreover, biaxial compressive strain modulates the H adsorption free energy (Δ*G*_H*_) toward zero, achieving almost thermoneutral H binding (Δ*G*_H*_ = 0.08 and 0.07 eV for Hg and Zn, respectively) at 3% compressive strain. These results are comparable to those reported in literature for benchmark catalysts, as summarized in Table S3 of Supporting Information, where we also have included the estimated theoretical overpotential derived from the Δ*G*_H_, calculated as η = |Δ*G*_H_|/e (where is the elementary charge).^74^ Our analysis reveals that H adsorption strength modulation stems primarily from electronic factors, such as the d-band center (ϵ_d_) position. Tensile (compressive) strain downshifts (upshifts) the d-band center of the late transition metal sites, unveiling an interesting pattern that aligns with the Δ*G*_H*_ changes, characteristic in transition metals with more than half-filled d orbitals. Furthermore, the pDOS analysis revealed that lattice stretching results in the depletion of the charge population on hybrid states, which contributes to the destabilization of the H-metal bonds, corroborated by ICOHP analysis. These results are supported by the evolution of the differential charge analysis, which shows an increase in charge accumulation between Hg­(Zn) and Pt atoms under lattice expansion, ultimately leading to more electrostatic repulsion, thus explaining the decrease in bonding strength. Our contribution explores strain engineering as an effective strategy to tailor the activity of 2D mineral-based catalysts for HER, advancing our understanding of how mechanical manipulation can effectively modulate the catalytic properties of these materials.

## Supplementary Material



## Notes

Charge difference plots for H adsorption on Pt sites; top views of adsorbed H as a function of coverage; tables with adsorption energies as a function of biaxial strain for Pt_2_HgSe_3_ and Pt_2_ZnSe_3_; distances between H and Hg/Zn sites and Bader charges as a function of strain; integrated COHP as a function of biaxial strain; comparative table with Δ*G*_H_ of previous reported promising catalysts for HER and optimized crystal structures of Pt_2_HgSe_3_ and Pt_2_ZnSe_3_ monolayers (PDF)
