# Supplementary material for: Strain-Engineered Jacutingaite Analogs as Efficient 2D Catalysts for Hydrogen Evolution Reactions
Source: ACS Omega. 2025 Nov 28;10(48):59469–77. doi: 10.1021/acsomega.5c09065 (PMC12771121; doi:10.1021/acsomega.5c09065)
Supplement: Supplementary file 1 [file ao5c09065_si_001.pdf]

Supplementary Information: Strain-Engineered Jacutingaite Analogs as Efficient 2D Catalysts for Hydrogen Evolution Reactions

Caique Campos de Oliveira and Pedro Alves da Silva Autreto*

Center for Natural and Human Sciences (CCNH), Federal University of ABC (UFABC), 09210-170, Santo André-SP, Brazil

E-mail: pedro.autreto@ufabc.edu.br

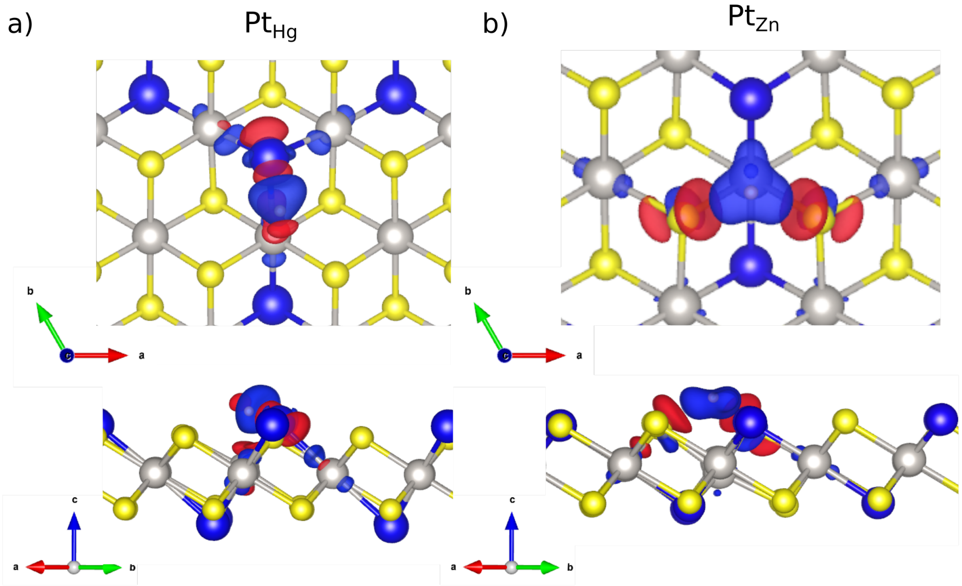

Figure S1: Top and side view of the charge difference for H adsorbed on a) Pt_{Hg} and Pt_{Zn} sites. Blue and red regions depict charge accumulation and depletion, respectively. Isosurfaces were set to 1.25×10^{-3} e/Å^3

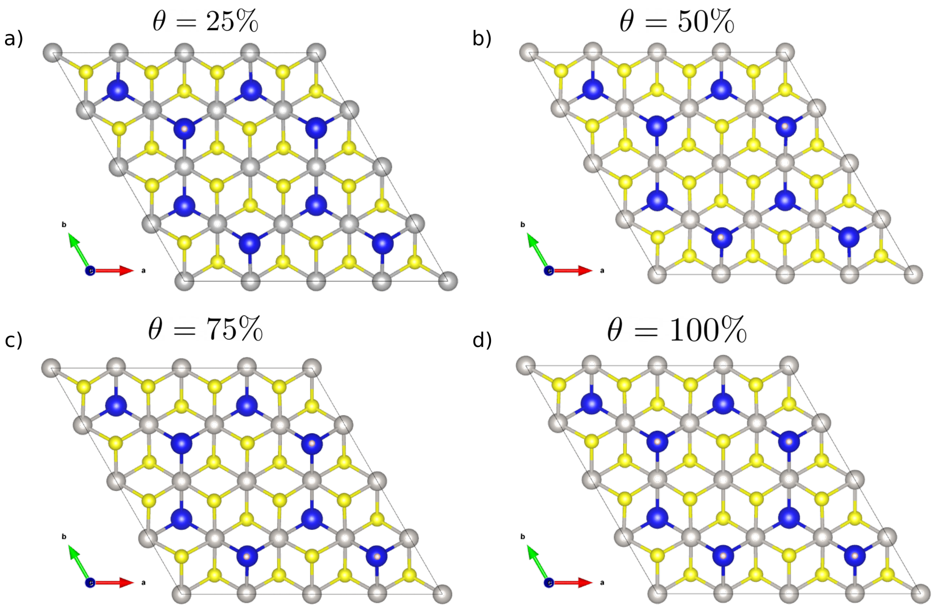

Figure S2: Top view of the optimized structures of H adsorbed on Pt_2XSe_3 for different coverages of a) $\theta = 25\%$, b) $\theta = 50\%$, c) $\theta = 75\%$ and d) $\theta = 100\%$.

Table S1: H adsorption as a function of biaxial strain for Pt_2HgSe_3.

ε (%)	E_{H*} (eV)	E_*	E_{ads} (eV)	ΔG_{H*}
-3	-211.9023447	-208.3602181	-0.16	0.07
-2	-212.2577511	-208.7718258	-0.11	0.13
-1	-212.4513255	-209.0210436	-0.05	0.19
0	-212.4793751	-209.0984331	0.00	0.24
1	-212.3538703	-209.0184013	0.05	0.29
2	-212.07520983	-208.77625979	0.08	0.32
3	-211.6547281	-208.3916352	0.12	0.36

Table S2: H adsorption as a function of the biaxial strain in Pt_2ZnSe_3.

ε (%)	E_{H*} (eV)	E_*	E_{ads} (eV)	ΔG_{H*}
-3	-220.8211366	-217.2653169	-0.17	0.07
-2	-221.1913744	-217.7011696	-0.11	0.13
-1	-221.3939526	-217.9653896	-0.05	0.19
0	-221.4302888	-218.0523136	0.00	0.24
1	-221.3030936	-217.9709675	0.05	0.29
2	-221.0233621	-217.7281911	0.09	0.33
3	-220.5930733	-217.3206687	0.11	0.35

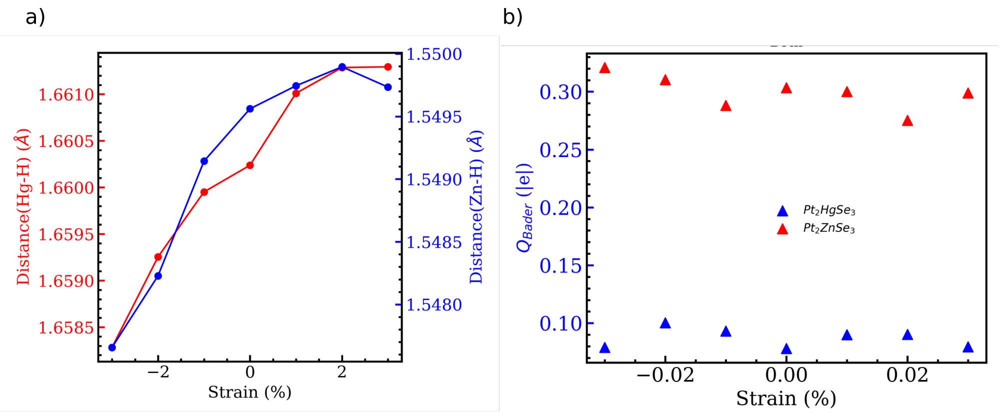

Figure S3: a) Distance between adsorbed H and metal (Hg and Zn) site. b) Bader charge on the adsorbed H as a function of biaxial strain.

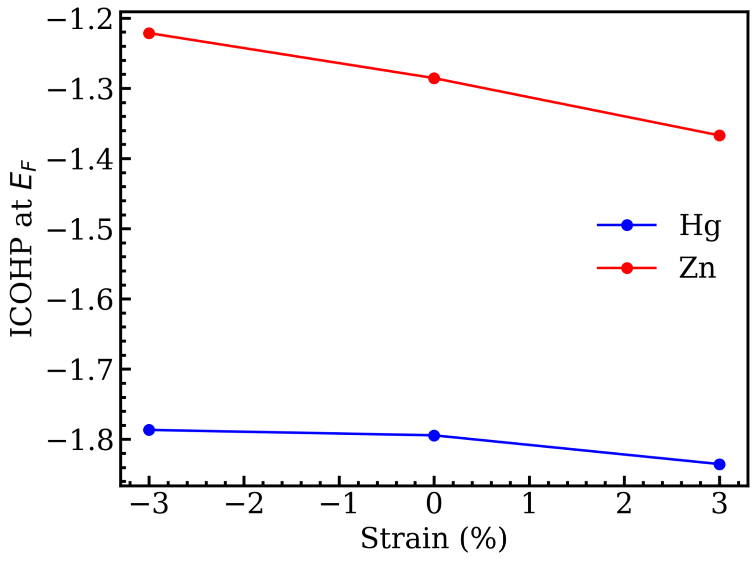

Figure S4: Integrated Crystal Orbital Hamilton Population (ICOHP) for Hg and Zn sites as a function of the strain.

Table S3: Compilation of benchmark and similar TMD materials catalytic activity in Hydrogen Evolution Reactions.

Material	ΔG_H (eV)	η_{Th} (V)	η_{10} (V)	Electrolyte	Ref.
Pt(111)	-0.09	0.09	0.09	-	[1]
2H-MoS_2	0.06	0.06	-	H_2SO_4 (pH = 0)	[2]
2H-MoS_2 (BP)	0.15	0.15	-	H_2SO_4 (pH = 0.2)	[3]
$PtSe_2$	0.01	0.01	0.015	H_2SO_4 (pH = 0)	[4]
Ru-MoTe_2	-0.01	-0.01	-	-	[5]
p-WTe_2	-0.17	0.17	-	-	[6]
MoS_2/ZnO	-0.04	0.04	-	-	[7]
Pt_2HgSe_3	0.08	0.08	-	-	This work
Pt_2ZnSe_3	0.07	0.07	-	-	This work

The optimized structures for Pt_2HgSe_3 and Pt_2ZnSe_3 are provided in POSCAR format below:

```
Pt2HgSe3
   1.0000000000000000
     3.7523302850450215   -6.4992267004773723    0.0000000000000000
     3.7523302850450215    6.4992267004773723   -0.0000000000000000
    -0.0000000000000000   -0.0000000000000000   23.4883299759743132
   Pt   Hg   Se
     4     2     6
Direct
  0.0000000000000000 -0.0000000000000000  0.4999999999986002
  0.0000000000017266  0.4999999999950191  0.4999999999986002
  0.4999999999950191  0.0000000000017266  0.4999999999986002
  0.4999999999967457  0.4999999999967457  0.4999999999986002
  0.3333333333271398  0.6666666666663517  0.4253897441682908
  0.6666666666663517  0.3333333333271398  0.5746102558333364
  0.3368412927207782  0.1684206463573693  0.4453137667870696
  0.1684206463573693  0.3368412927207782  0.5546862332145575
  0.1684206463590959  0.8315793536343877  0.5546862332145575
  0.8315793536343877  0.1684206463590959  0.4453137667870696
  0.8315793536361143  0.6631587072727061  0.4453137667870696
  0.6631587072727061  0.8315793536361143  0.5546862332145575
```

```
Pt2ZnSe3
   1.0000000000000000
     3.7313666217222017   -6.4629139636576038    0.0000000000000172
```

```
    3.7313666217257300    6.4629139636644197   -0.0000000000000161
    0.0000000000000255   -0.0000000000000525   20.7703534230720948
  Pt    Zn    Se
    4     2     6
Direct
 -0.0000000000000000  0.0000000000000000  0.4999996150421424
  0.9999998989594303  0.4999997261871556  0.4999996150421424
  0.4999997261871556  0.9999998989594303  0.4999996150421424
  0.4999996251465859  0.4999996251465859  0.4999996150421424
  0.3333330160706822  0.6666662342224967  0.4345044111109417
  0.6666662342224967  0.3333330160706822  0.5654948189791726
  0.3357826516453958  0.1678912749967979  0.4371140098423055
  0.1678912749967979  0.3357826516453958  0.5628852202470767
  0.1678911745597277  0.8321080757346931  0.5628852202470767
  0.8321080757346931  0.1678911745597277  0.4371140098423055
  0.8321079752842363  0.6642165986338885  0.4371140098423055
  0.6642165986338885  0.8321079752842363  0.5628852202470767
```

References

(1) Nørskov, J. K.; Bligaard, T.; Logadottir, A.; Kitchin, J. R.; Chen, J. G.; Pandelov, S.; Stimming, U. Trends in the Exchange Current for Hydrogen Evolution. *Journal of The Electrochemical Society* **2005**, *152*, J23.

(2) Hinnemann, B.; Moses, P. G.; Bonde, J.; Jørgensen, K. P.; Nielsen, J. H.; Horch, S.; Chorkendorff, I.; Nørskov, J. K. Biomimetic hydrogen evolution: MoS2 nanoparticles as catalyst for hydrogen evolution. *Journal of the American Chemical Society* **2005**, *127*, 5308–5309.

(3) Li, H.; Tsai, C.; Koh, A. L.; Cai, L.; Contryman, A. W.; Fragapane, A. H.; Zhao, J.; Han, H. S.; Manoharan, H. C.; Abild-Pedersen, F.; Nørskov, J. K.; Zheng, X. Activating and optimizing MoS2 basal planes for hydrogen evolution through the formation of strained sulphur vacancies. *Nature Materials* **2016**, *15*, 48–53.

(4) Jeon, H.; Kwon, H. J.; Lee, J.; Han, S. K.; Kim, H.; Heo, J.; Han, J.; Shin, S.; Park, J.; Cho, M. K.; Preston, D. J.; Kim, I. S.; Kim, M.; Lee, W.-K. Strain-Enabled Band Structure Engineering in Layered PtSe2 for Water Electrolysis under Ultralow Overpotential. *ACS Nano* **2025**, *19*, 9107–9120, PMID: 40012087.

(5) Huang, Y.; Chen, Y.; Zhang, Y.; Zhang, H.; Xiong, K.; Ye, X.; Liu, Q.; Zhu, J. Transition-metal atoms embedded MoTe2 single-atom catalyst for efficient electrocatalytic hydrogen evolution reaction. *Applied Surface Science* **2025**, *680*, 161335.

(6) Huang, H.; Hu, G.; Hu, C.; Fan, X. Enhanced hydrogen evolution reactivity of T'-phase tungsten dichalcogenides (WS2, WSe2, and WTe2) materials: A DFT study. *International Journal of Molecular Sciences* **2022**, *23*, 11727.

(7) Yang, J.; Li, X.; Yang, Y.; Dou, R. Strain Engineering the Optoelectronic and HER Behavior of MoS2/ZnO Heterojunction: A DFT Investigation. *The Journal of Physical Chemistry Letters* **2025**, *16*, 2731–2741, PMID: 40051187.
